# The efficacy and safety of cannabidiol (CBD) in pediatric patients with Dravet Syndrome: a narrative review of clinical trials

**DOI:** 10.1186/s40001-024-01788-6

**Published:** 2024-03-18

**Authors:** Nicholas Aderinto, Gbolahan Olatunji, Emmanuel Kokori, Yusuf Ismaila Ajayi, Olumide Akinmoju, Abiola Samuel Ayedun, Oluwapelumi Ikeoluwa Ayoola, Noah Oluwaseun Aderinto

**Affiliations:** 1 Department of Medicine and Surgery, Ladoke Akintola University Teaching Hospital, Ogbomoso, Nigeria; 2 Department of Medicine and Surgery, University of Ilorin, Ilorin, Nigeria; 3 Department of Medicine and Surgery, Obafemi Awolowo University Teaching Hospital, Ife, Nigeria; 4 Department of Medicine and Surgery, University of Ibadan, Ibadan, Nigeria; 5 Department of Medicine, University of Abuja, Abuja, Nigeria; 6 Bowen University, Iwo, Nigeria; 7 Department of Paediatrics and Child Health, UNIOSUN Teaching Hospital, Osogbo, Nigeria; 8 Department of Medicine and Surgery, Ladoke Akintola University of Technology, PMB 5000, Ogbomosho, Nigeria

**Keywords:** Dravet Syndrome, Cannabidiol (CBD), Pediatric patients, Seizure frequency, Antiepileptic drugs

## Abstract

**Background:**

Dravet Syndrome (DS) is a rare and severe form of childhood epilepsy that is often refractory to conventional antiepileptic drugs. Emerging evidence suggests that Cannabidiol (CBD) offer therapeutic benefits for DS. This review aims to evaluate the efficacy and safety of CBD in pediatric patients with DS based on data from ten clinical trials.

**Methods:**

A review was conducted to identify clinical trials assessing the efficacy and safety of CBD in pediatric patients diagnosed with DS. PubMed, MEDLINE, Scopus, Web of Science, and relevant grey literature were systematically searched for relevant articles up to October 2023, and clinical trials within the last 10 years were included. The search strategy incorporated controlled vocabulary terms and keywords related to "Cannabidiol," "Dravet Syndrome," and "pediatric patients."

**Results:**

The analysis revealed promising efficacy outcomes. Notably, CBD demonstrated substantial reductions in seizure frequency, with some patients achieving seizure freedom. The findings emphasised the consistency of CBD's efficacy across different patient subgroups. The safety profile of CBD was generally acceptable, with adverse events often being manageable.

**Conclusion:**

This review consolidates evidence from multiple clinical trials, affirming the potential of CBD as a promising treatment option for pediatric patients with DS. While further research is needed to address existing knowledge gaps, CBD's efficacy and acceptable safety profile make it a valuable addition to the therapeutic tools for DS.

## Introduction

Dravet Syndrome (DS), also known as Severe Myoclonic Epilepsy of Infancy (SMEI), is a rare and debilitating form of epilepsy characterised by recurrent febrile and afebrile seizures, ataxia, cognitive impairment, and developmental delays [1, 2]. Onset typically occurs in early infancy and imposes a substantial burden on the quality of life for affected individuals [3]. It is primarily caused by mutations in the SCN1A gene, leading to neuronal hyperexcitability and intractable seizures [4]. Additionally, patients with DS are at risk of sudden unexplained death, making early and effective seizure control crucial [5].

The genetic basis of DS is largely attributed to heterozygous mutations in the NaV1.1 alpha subunit of voltage-gated sodium ion channels encoded by the SCN1A gene [6, 7]. These mutations result in the loss of function of NaV1.1 channels, which is critical for normal brain function and leads to seizures and epilepsy [8, 9]. In some cases, these pathogenic SCN1A variants can be inherited, while in others, de novo mutations occur [10, 11]. Other genes like SCN1B, GABRA1, PCDH19, GABRG2, HCN1, and STXBP1 have also been implicated in DS, although not all cases are genetic, and not all genetic mutations result in DS [11].

DS typically manifests in the first year of life, often with a normal early childhood development followed by the onset of seizures around 4–12 months of age [12]. Seizures can be medically refractory, leading to recurrent status epilepticus and various comorbidities, including intellectual disability, ataxia, and an increased risk of early mortality [13]. Therefore, the impact of DS on affected individuals is profound, encompassing not only seizures but also developmental and cognitive challenges [14–16]. Despite several decades of research, current treatment options for DS remain limited, often necessitating the use of polypharmacy with antiepileptic drugs [17]. Medications like sodium valproate, topiramate, and stiripentol are commonly used, but some, like carbamazepine, should be avoided [18]. Recent studies have demonstrated the efficacy of stiripentol in managing seizures associated with DS [19, 20]. Notably, stiripentol has shown potential as an additional therapy, offering a new avenue for improving seizure control in individuals with this challenging condition [21]. Additionally, dietary therapies, such as the ketogenic diet, and non-pharmacologic strategies, like avoiding seizure triggers, are considered [22]. In addition, emerging evidence suggests that other antiepileptic drugs with sodium channel-blocking properties, such as oxcarbazepine and lamotrigine, also pose a risk of exacerbating seizures in individuals with DS. While these interventions can reduce seizure frequency and disease severity, they do not address the underlying pathogenesis.

Cannabidiol (CBD), a non-psychoactive compound derived from the cannabis plant, has garnered attention as a potential treatment for DS [23–25]. Clinical trials, along with the FDA's approval of Epidiolex for DS and Lennox–Gastaut Syndrome, highlight CBD's potential as an alternative therapy [26, 27]. Existing studies often focus on specific aspects of the potential treatment, such as seizure reduction or safety profiles, rather than providing a holistic view of CBD's efficacy and safety in managing the multifaceted challenges of DS [28, 29].

While the predominant emphasis in the literature lies in investigating the impact of CBD on convulsive seizures in pediatric patients with DS. Studies have begun to shed light on the potential efficacy of CBD in mitigating various seizure manifestations beyond convulsions, such as absence seizures and myoclonic seizures [30, 31]. Moreover, CBD has demonstrated a well-documented interaction with clobazam. Studies have consistently reported that co-administration of CBD and clobazam can lead to alterations in the pharmacokinetics of both substances [32, 33]. This interaction highlights the necessity for close monitoring and potential dosage adjustments when utilising CBD alongside other anti-epileptics in the treatment of DS. This review aims to examine the existing body of evidence regarding the efficacy and safety of CBD in the management of DS, considering the limitations of current treatment options and the potential benefits of CBD-based therapies.

## Methodology

### Literature search strategy

**Fig. 1:**
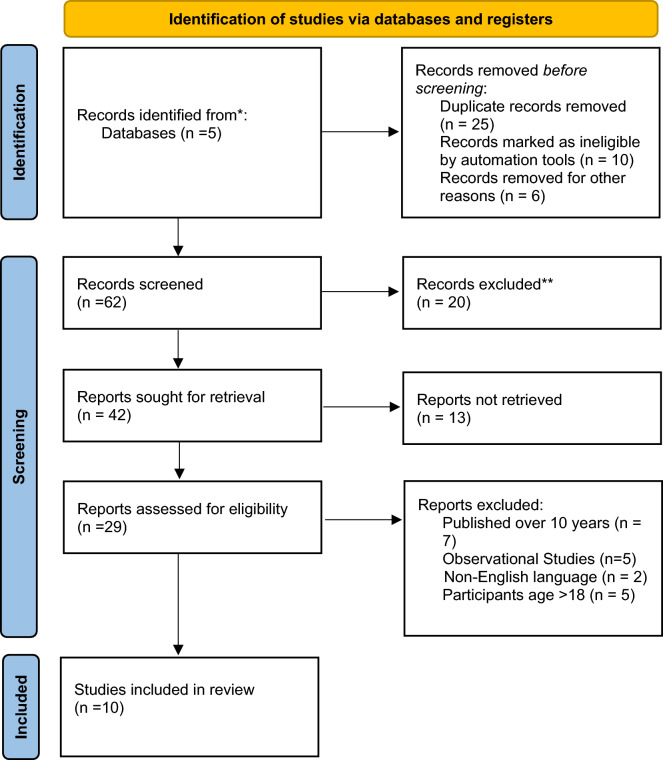
Literature search strategy

In this study, an extensive search was conducted in PubMed, MEDLINE, Scopus, Web of Science, and relevant grey literature. The search incorporated a range of search terms, such as "cannabidiol," "CBD," "Dravet Syndrome," "seizures," and related keywords. Studies published from the inception of each database until the present were included in the search. The search was specifically limited to articles published in the English language. See Fig. 1.

### Inclusion and exclusion criteria

For inclusion, the study must be a clinical trial, published within the last 10 years to provide the latest evidence, published in the English language and focused on the use of CBD in the management of DS in individuals aged 18 or less. This review specifically looked at studies that addressed seizure reduction, safety profiles, and broader impacts on DS. Animal studies, meta-analyses, reviews, and observational studies were excluded.

### Data extraction

For each selected study, relevant information, such as study design, sample size, patient demographics, CBD dosing regimen, treatment duration, outcomes measured, and reported results, was extracted. Two reviewers carried out the data extraction process independently, and any discrepancies were resolved through discussion and, if necessary, consultation with a third reviewer.

### Data synthesis and analysis

A narrative synthesis approach was employed to summarise and analyse the findings of the selected studies. The effectiveness of CBD in reducing seizures, its impact on cognitive and developmental outcomes, and its safety profile were subject to thorough examination. Heterogeneity in study designs, patient populations, and dosing regimens was carefully considered when concluding, and any inconsistencies or discrepancies in the literature were brought to attention.

## Results

This study reviewed data from ten distinct clinical trials. See Table 1. These interventions varied in terms of the type of CBD administered, which could be a pharmaceutical formulation or plant-derived. The doses of CBD ranged from 2 to 20 mg/kg/d, with varying treatment durations spanning from 4 to 72 weeks. Additionally, the frequency of administration varied, with some studies utilising once-daily dosing and others opting for twice-daily schedules. The duration of follow-up showed significant diversity across these studies, extending from 4 to 72 weeks. In total, these ten studies included 1,724 participants. On average, each study featured approximately 172.4 participants. CBD doses ranged from a minimum of 2.5 mg/kg/day to 30 mg/kg/day, with preparations typically being highly purified CBD in a 100 mg/ml oral solution, and the mean modal dose across the studies was approximately 22 mg/kg/day. The primary efficacy outcomes were evaluated based on the percentage reduction in convulsive and total seizures. Furthermore, clinical improvement was assessed through the Subject/Clinician Global Impression of Change (SCGIC) scale, and quality of life was measured using the Childhood Epilepsy Questionnaire. The reduction in seizure frequency for convulsive seizures ranged from 38 to 74%, and for total seizures, it varied from 40 to 84%. SCGIC scale reported improvements in the range of 81–84%. In addition, 24-h ambulatory EEG was utilised to monitor EEG spikes, aligning these outcome measures with those commonly employed to assess the efficacy of other antiepileptic drugs (AEDs).

**Table 1:** Characteristics of included studies

Author/year	Study design	Age	Dosage of CBD	Study population	Duration of treatment	Concomitant medication/comparisons	Efficacy outcomes	Safety outcomes
Iannone LF et al., 2021	Randomized Open-Label Extension Trial	Mean age: 17.0 ± 13.1	Thirty centers were enrolled from December 2018 to December 2019 within the open-label prospective EAP up to a maximum of 25 mg/kg per day	93	1 year	CBD was mostly coadministered with valproic acid (62.2%) and clobazam (41.5%)	At 3-month follow-up, compared to the 28-day baseline period, the percentage of patients with at least a 50% reduction in seizure frequency was 40.2% (plus 1.2% seizure-free). Retention rate was similar according to diagnosis, while we found an increased number of patients remaining under treatment in the adult group	In the safety dataset, 29 (31.2%) dropped out: reasons were lack of efficacy [16 (17.2%)] and adverse events (AEs) [12 (12.9%)], and one met withdrawal criteria (1.1%). Most reported AEs were somnolence (22.6%) and diarrhea (11.9%), followed by transaminase elevation and loss of appetite
Devinsky O et al., 2021	Double-blind RCT	4–10 years	20 mg/kg/CBD and placebo	34	The double-blind trial comprised 4-week baseline, 3-week treatment (including titration), 10-day taper, and 4-week follow-up periods	Multiple pharmacokinetic blood samples were taken on the first day of dosing and at end of treatment for measurement of CBD, its metabolites 6-OH-CBD, 7-OH-CBD, and 7-COOH-CBD, and antiepileptic drugs (AEDs; clobazam and metabolite *N*-desmethylclobazam [N-CLB], valproate, levetiracetam, topiramate, and stiripentol)	CBD did not affect concomitant AED levels, apart from increased N-CLB (except in patients taking stiripentol)	The most common AEs on CBD were pyrexia, somnolence, decreased appetite, sedation, vomiting, ataxia, and abnormal behaviour. Six patients taking CBD and valproate developed elevated transaminases; none met criteria for drug-induced liver injury and all recovered. No other clinically relevant safety signals were observed
Scheffer, Ingrid E., et al., 2021	Open label extension trial	2–18 years	Mean modal dose of 22 mg/kg/day;	330	Median treatment duration was 444 days	84% were on concomitant valproic acid	In patients from GWPCARE1 Part B and GWPCARE2, the median reduction from baseline in monthly seizure frequency assessed in 12-week periods up to Week 156 was 45–74% for convulsive seizures and 49–84% for total seizures. Across all visit windows, ≥ 83% patients/caregivers completing a Subject/Caregiver Global Impression of Change scale reported improvement in overall condition	Adverse events (AEs) occurred in 97% patients (mild, 23%; moderate, 50%; severe, 25%). Commonly reported AEs were diarrhea (43%), pyrexia (39%), decreased appetite (31%), and somnolence (28%). Twenty-eight (9%) patients discontinued due to AEs. Sixty-nine (22%) patients had liver transaminase elevations > 3 × upper limit of normal
Ian Miller et al., 2020	Double-blind, placebo-controlled, randomised clinical trial	2 to 18 years	Pharmaceutical formulation of cannabidiol, 10 and 20 mg/kg/d, vs placebo	199	14 weeks	Placebo	Of 198 eligible patients (mean [SD] age, 9.3 [4.4] years; 104 female [52.5%]), 66 were randomised to the CBD10 group, 67 to the CBD20 group, and 65 to the placebo group, and 190 completed treatment. The percentage reduction from baseline in convulsive seizure frequency was 48.7% for CBD10 group and 45.7% for the CBD20 group vs 26.9% for the placebo group; the percentage reduction from placebo was 29.8% (95% CI 8.4–46.2%; *P* = .01) for CBD10 group and 25.7% (95% CI 2.9–43.2%; *P* = .03) for the CBD20 group	The most common adverse events were decreased appetite, diarrhea, somnolence, pyrexia, and fatigue. Five patients in the CBD20 group discontinued owing to adverse events. Elevated liver transaminase levels occurred more frequently in the CBD20 (*n* = 13) than the CBD10 (*n* = 3) group, with all affected patients given concomitant valproate sodium
Jonathan Halford et al., 2019	RCT	Mean age: 9.8 years	Patients received GW’s plant-derived pharmaceutical formulation of highly purified CBD (100 mg/mL) in oral solution	289	72 weeks	Patients were taking a median of three concurrent antiepileptic drugs with 68% taking clobazam, 63% valproate, and 39% stiripentol	Median % reductions from baseline assessed in 12-week intervals were 44%–57% for convulsive and 49–67% for total seizures through 72 weeks. Over 80% of patients/caregivers reported improvements in overall condition	Adverse events (AEs) and serious AEs were reported by 96% and 32% of patients; 7% discontinued due to AEs. Elevated liver transaminases > 3 × upper limit of normal were reported in 9% of patients; none had severe liver injury. Two nontreatment-related deaths were reported
Linda C. Laux et al., 2019	RCT	607 Children and adults with LGS/DS taking stable doses of antiepileptic drugs	Mean age: 12.8	607	96 weeks		Of the 607 patients in the SAS, 58 had DS and 94 had LGS (*N* = 152); 455 patients had other TREs. Twenty-eight percent of LGS/DS patients withdrew, primarily owing to lack of efficacy (20%). LGS/DS patients were taking a median of 3 (0–10) concomitant AEDs. Median treatment duration was 78.3 (range, 4.1–146.4) weeks. Between weeks 12 and 96, median CBD dose ranged from 21 to 25 mg/kg/day. At 12 weeks, add-on CBD reduced median monthly major motor seizures by 50% and total seizures by 44%, with consistent reductions in both seizure types through 96 weeks. At 12 weeks, the proportions of patients with ≥ 50%, ≥ 75%, and 100% reductions in major motor seizures were 53%, 23%, and 6%; the proportions with corresponding reductions in total seizures were 46%, 26%, and 5%. Responder rates for both seizure types were consistent through 96 weeks. CBD had an acceptable safety profile	The most common AEs were somnolence (30%) and diarrhea (24%)
Devinsky O et al., 2019	Randomized Open-Label Extension Trial	Mean: 9.8 (4.4)	Pharmaceutical formulation of highly purified CBD in oral solution (100 mg/mL), titrated from 2.5 to 20 mg/kg/d over a 2‐week period	278	48 weeks	Twenty‐two of the 128 patients from GWPCARE1 (17.2%), all taking valproic acid, had liver transaminase elevations ≥ 3 times the upper limit of normal	In patients from GWPCARE1 Part B, the median reduction from baseline in monthly seizure frequency assessed in 12‐week periods up to week 48 ranged from 38 to 44% for convulsive seizures and 39% to 51% for total seizures. After 48 weeks of treatment, 85% of patients/caregivers reported improvement in the patient's overall condition on the Subject/Caregiver Global Impression of Change scale	Commonly reported AEs were diarrhea (34.5%), pyrexia (27.3%), decreased appetite (25.4%), and somnolence (24.6%). Seventeen patients (6.4%) discontinued due to AEs. Seventeen patients (6.4%) discontinued due to AEs
Bláthnaid McCoy et al., 2018	RCT	Mean age: 10.15 years	The dose ranged from 2 to 16 mg/kg/day of CBD and 0.04 to 0.32 mg/kg/day of THC	20	20 weeks		Nineteen participants completed the 20-week intervention. Mean dose achieved was 13.3 mg/kg/day of CBD (range 7–16 mg/kg/day) and 0.27 mg/kg/day of THC (range 0.14–0.32 mg/kg/day). There was a statistically significant improvement in quality of life, reduction in EEG spike activity, and median motor seizure reduction of 70.6%, with 50% responder rate of 63%	Adverse events, common during titration included somnolence, anorexia, and diarrhea. Abnormalities of liver transaminases and platelets were observed with concomitant valproic acid therapy
Orrin Devisky et al., 2018	Double-blind RCT	4–10 years	CBD (5, 10, or 20 mg/kg/d) or placebo taken twice daily	34	4-week baseline, 3-week treatment (including titration), 10-day taper, and 4-week follow-up periods		Exposure to CBD and its metabolites was dose-proportional (AUC0–t). CBD did not affect concomitant AED levels, apart from an increase in N-CLB (except in patients taking stiripentol)	The most common AEs on CBD were pyrexia, somnolence, decreased appetite, sedation, vomiting, ataxia, and abnormal behaviour. Six patients taking CBD and valproate developed elevated transaminases; none met criteria for drug-induced liver injury and all recovered. No other clinically relevant safety signals were observed
Orrin Devisky et al., 2017	Double-blind RCT	The mean age of the patients was 9.8 years	Cannabidiol oral solution at a dose of 20 mg per kilogram of body weight per day or placebo	120	14-week treatment period	Placebo	The median frequency of convulsive seizures per month decreased from 12.4 to 5.9 with cannabidiol, as compared with a decrease from 14.9 to 14.1 with placebo (adjusted median difference between the cannabidiol group and the placebo group in change in seizure frequency, − 22.8 percentage points; 95% confidence interval [CI], − 41.1 to − 5.4; *P* = 0.01). The percentage of patients who had at least a 50% reduction in convulsive seizure frequency was 43% with cannabidiol and 27% with placebo (odds ratio, 2.00; 95% CI 0.93 to 4.30; *P* = 0.08). The frequency of total seizures of all types was significantly reduced with cannabidiol (*P* = 0.03), but there was no significant reduction in nonconvulsive seizures. The percentage of patients who became seizure-free was 5% with cannabidiol and 0% with placebo (*P* = 0.08)	Adverse events that occurred more frequently in the cannabidiol group than in the placebo group included diarrhea, vomiting, fatigue, pyrexia, somnolence, and abnormal results on liver-function tests. There were more withdrawals from the trial in the cannabidiol group

### Efficacy outcomes

The analysis of the ten clinical trials provides a profound understanding of the efficacy of CBD in pediatric patients with DS. Iannone et al. conducted a randomised open-label extension trial [34]. Their findings show that CBD at 25 mg/kg per day had a remarkable impact. At the 3-month follow-up, 40.2% of patients substantially reduced seizure frequency, with 1.2% experiencing seizure freedom. A particularly interesting aspect is the observed stability in patient retention across the diagnosis spectrum, suggesting the potential for CBD's consistent efficacy. This study also revealed that CBD's efficacy remained independent of the dosage used, which has implications for treatment optimisation.

Devinsky et al. study demonstrated a 48.7% reduction in convulsive seizure frequency and a 45.7% reduction in total seizure frequency [23]. Notably, the findings indicated that CBD at 20 mg/kg/ per day did not significantly influence concomitant antiepileptic drug (AED) levels, reinforcing its efficacy as an independent therapeutic agent. The open-label extension trial by Scheffer et al. highlighted the sustainability of CBD's efficacy at 22 mg/kg per day [18]. Patients in this study experienced sustained, clinically meaningful reductions in seizure frequency. After 12 weeks, add-on CBD treatment led to a 50% reduction in median monthly major motor seizures and a 44% reduction in total seizures. Moreover, the study reported that 83% or more of patients or caregivers noted an improvement in their overall condition. The inclusion of patients taking concomitant valproic acid provided valuable insights into CBD's potential as a long-term treatment option for those with DS.

Miller et al. embarked on a double-anonymized, placebo-controlled, randomised clinical trial involving pediatric patients aged 2 to 18 [28]. The study underscored the improved safety and tolerability profile of a 10-mg/kg/d CBD dosage, significantly advancing in treating children with treatment-resistant DS. In a study conducted by Halford et al. involving patients with an average age of 9.8 years, significant reductions in convulsive and total seizures were reported with CBD of 100 mg/mL in oral solution [35]. While over 80% of patients or caregivers noted improvements in their overall condition, it underscores the substantial enhancement in the quality of life for DS patients.

Linda et al. reported substantial reductions in major motor and total seizures with 10 mg/kg per day of CBD [24]. Devinsky et al. reported statistically significant reductions in convulsive and total seizure frequency with 20 mg/kg per day of CBD [36]. Additionally, the study noted improvements in Subject/Caregiver Global Impression of Change (S/CGIC) scores, demonstrating CBD's positive impact on seizure control and patients' overall well-being. Similarly, Bláthnaid et al. reported a statistically significant improvement in quality of life, a median motor seizure reduction of 70.6%, and a 50% responder rate of 63%, emphasising the transformative potential of CBD at 2 to 16 mg/kg per day in enhancing the lives of young patients with DS [22].

Devinsky et al. noted that CBD at 20 mg/kg per day led to a more substantial reduction in convulsive seizure frequency compared to a placebo [29]. However, it is important to recognise that this increased efficacy was associated with higher rates of adverse events, underscoring the importance of balancing therapeutic benefits with potential risks. In Devinsky et al., the study leading to FDA approval, the findings pointed to a statistically significant reduction in the median frequency of convulsive seizures per month with CBD at 20 mg/kg per day, reaffirming the potential of this treatment in effectively reducing seizure frequency in patients with DS [23].

In terms of efficacy, a noteworthy observation is the absence of substantial distinctions among different dosages, 5 mg/kg/day, 10 mg/kg/day, and 20 mg/kg/day, compared to the placebo [23, 28, 34]. All treatment groups exhibited considerable enhancements in reducing seizure frequency relative to the placebo; however, discernible variations between these dosage tiers were notably limited.

### Safety outcomes

Ensuring the safety of CBD in pediatric patients with DD is paramount. Each of the reviewed clinical trials provides valuable insights into the safety profile of CBD in this patient population. The randomised open-label extension trial conducted by Iannone et al. reported that 31.2% of patients dropped out for various reasons. Common adverse events included somnolence (22.6%), diarrhoea (11.9%), transaminase elevation, and loss of appetite [34]. Notably, only 1.1% of patients met withdrawal criteria. In the case of Devinsky et al., the study identified common adverse events associated with CBD, including pyrexia, somnolence, decreased appetite, sedation, vomiting, ataxia, and abnormal behaviour [23]. Intriguingly, six patients taking CBD and valproate experienced elevated transaminases, but none met the criteria for drug-induced liver injury, and all patients eventually recovered. The study indicated that lethargy is particularly common in patients taking CBD alongside clobazam. The study emphasised that exposure to CBD and its metabolites increases proportionally with the dose.

The open-label extension trial by Scheffer et al. reported that adverse events occurred in 97% of patients, with the majority being mild (23%) or moderate (50%) [18]. Commonly reported adverse events included diarrhoea (43%), pyrexia (39%), decreased appetite (31%), and somnolence (28%). Importantly, 9% of patients experienced liver transaminase elevations greater than three times the upper limit of normal, although none of these cases led to severe liver injury. For Miller et al. specific safety data with percentages were not provided [28]. However, the study emphasised that long-term add-on CBD treatment for DS was generally well tolerated, with an adverse event profile similar to that observed in controlled trials. Similarly, for Halford et al. specific safety data with percentages were unavailable [35]. Nevertheless, it was reported that long-term treatment with add-on CBD in patients with DS produced sustained seizure reductions with no new safety concerns.

The data provided by Linda et al. did not specify the percentages of adverse events [24]. Nonetheless, it was noted that CBD had an acceptable safety profile. Safety data from this study emphasised the overall tolerability of CBD in patients with DS. Miller et al. reported that long-term add-on CBD treatment for DS was generally well tolerated [36]. McCoy et al. reported that adverse events common during titration included somnolence, anorexia, and diarrhoea [22]. Abnormalities of liver transaminases and platelets were observed with concomitant valproic acid therapy. Nevertheless, this THC-containing cannabinoid preparation was generally considered safe and well-tolerated.

## Discussion

The review of data from ten distinct clinical trials provides valuable insights into the use of CBD in pediatric patients with DS. These studies varied in terms of CBD type, dosage, treatment duration, and frequency of administration, yet they collectively shed light on the potential of CBD for managing DS.

The variability in CBD interventions across these studies presents challenges and opportunities for future research and clinical practice. While this diversity reflects real-world clinical scenarios, it complicates determining optimal treatment regimens. Whether pharmaceutical or plant-derived, the type of CBD administered could impact efficacy and safety. Moreover, the wide range of CBD doses and treatment durations underscores the need for further investigation into the most effective and sustainable treatment protocols. Additionally, the diverse frequency of administration across studies prompts whether once-daily or twice-daily dosing is more advantageous. Further exploration in this area could provide valuable guidance for treatment optimisation.

The collective findings from the ten clinical trials investigating the efficacy of CBD in pediatric patients with DS provide compelling insights into the potential of CBD as a therapeutic intervention. These trials consistently revealed notable reductions in both convulsive and total seizures, with some achieving remarkable results. For instance, Iannone et al. noted a 40.2% reduction in seizure frequency, with 1.2% of patients experiencing seizure freedom, while Devinsky et al. reported a 48.7% reduction in convulsive seizure frequency and a 45.7% reduction in total seizure frequency [23, 34].

These outcomes suggest a promising avenue for treatment. However, there is a pressing need for long-term studies to assess the sustained efficacy and safety of CBD in DS patients. While short-term results are encouraging, understanding the effects of extended CBD treatment is crucial. Additionally, future research must identify optimal dosages, as personalised dosing strategies could enhance treatment outcomes and minimise potential risks. Comparative studies that assess CBD's efficacy in comparison to other treatments or in conjunction with standard antiepileptic drugs (AEDs) can provide additional insights into its role in the treatment landscape. Furthermore, investigating how genetic and clinical factors influence individual responses to CBD treatment is vital. Identifying potential predictors of treatment outcomes can facilitate treatment customisation and improve overall efficacy and safety.

Safety considerations are paramount when assessing the use of CBD in pediatric patients with DS. The trials revealed that while CBD holds promise as a therapeutic intervention, it is not without adverse effects. Common adverse events reported across the trials included somnolence, diarrhoea, pyrexia, decreased appetite, vomiting, ataxia, sedation, and abnormal behaviour. Although relatively common, these adverse events were generally mild to moderate in intensity. Notably, a fraction of patients experienced elevated liver transaminases, albeit without severe liver injury, emphasising the importance of vigilant monitoring for potential liver-related adverse events. Importantly, the dropout rates due to adverse events in these trials were generally low, suggesting that most patients could tolerate CBD treatment. The trials also highlighted that while adverse events were observed, most patients did not meet withdrawal criteria, indicating an overall favourable risk–benefit profile.

These trials' results underscore CBD's promising role in managing DS, providing hope for improved seizure management and quality of life. However, the variability in CBD interventions and the occurrence of adverse events necessitate further investigation. Future research should determine the most effective treatment regimens, considering the type, dose, duration, and frequency of CBD administration. Long-term effects and interactions with other antiepileptic medications also require thorough examination. These findings hold practical significance for clinicians managing pediatric DS patients, emphasising the need for individualised treatment plans and close monitoring for adverse events. CBD-based therapies offer a valuable addition to the existing treatment options for DS, potentially improving patient outcomes and quality of life.

## Limitations of review

This review, which analysed data from ten distinct clinical trials involving many pediatric patients with DS, offers valuable insights into the efficacy and safety of CBD treatment. However, it is essential to acknowledge several limitations inherent to this review. The review's restriction to English-language studies poses a notable limitation. By focusing exclusively on English-language research, there is a risk of missing out on valuable non-English literature. This could introduce a language bias, potentially excluding relevant findings from studies conducted in other languages. Also, this review concentrated on clinical trials, thereby excluding observational studies. Despite these limitations, this systematic review offers valuable insights into CBD's potential benefits in managing DS. The synthesis of evidence and clinical implications outlined in the review provides a strong foundation for further research and clinical decision-making.

## Conclusion

This review offers a comprehensive and in-depth analysis of the existing evidence on the efficacy and safety of CBD in pediatric patients diagnosed with DS. The findings, compiled from ten distinct clinical trials, consistently point to the potential of CBD as a valuable therapeutic option for managing DS. Notably, CBD remarkably reduces seizure frequency and enhances the overall quality of life for affected patients. One of the most intriguing findings is the consistent efficacy of CBD across various studies, irrespective of the dosage administered. This suggests that CBD holds promise as a treatment that can deliver reliable results for a broad spectrum of DS patients. However, it is crucial to underscore the critical balance between its increased efficacy in some cases and the higher occurrence of adverse events. This balance reinforces the need for a cautious and individualised approach to treatment, ensuring that the therapeutic benefits outweigh potential risks.

The results of this review have significant implications for clinical practice, research endeavours, and healthcare policies. Clinicians managing pediatric patients with DS should consider CBD as a valuable adjunct therapy, particularly for cases refractory to other treatments. However, it is imperative to stay updated with evolving research and best practices to optimise CBD treatment regimens. While this review sheds light on the potential of CBD in transforming the management of DS, it also emphasises the need for further research. Well-designed clinical trials are warranted to refine treatment protocols, explore the optimal CBD dosage, and assess the durability of its therapeutic effects. Addressing long-term safety concerns, especially when CBD is used in conjunction with other antiepileptic drugs, is crucial to ensure the well-being of DS patients. Future research should delve deeper into the underlying mechanisms of CBD's antiseizure effects and its potential interactions with other medications. This will enhance our understanding of CBD's role in DS management and open new avenues for therapeutic innovation.

## Data Availability

Data sharing is not applicable to this article as no datasets were generated or analysed during the current study.

## Abbreviations

AEDs: Antiepileptic drugs
CBD: Cannabidiol
DS: Dravet Syndrome
EEG: Electroencephalogram
FDA: Food and Drug Administration
SCN1A: Sodium Voltage-Gated Channel Alpha Subunit 1
SMEI: Severe Myoclonic Epilepsy of Infancy
